# Mechanisms Underlying Sex Differences in Temporomandibular Disorders and Their Comorbidity with Migraine

**DOI:** 10.3390/brainsci14070707

**Published:** 2024-07-15

**Authors:** Adnan Khan, Sufang Liu, Feng Tao

**Affiliations:** Department of Biomedical Sciences, Texas A&M University School of Dentistry, Dallas, TX 75246, USA; akhan92@tamu.edu (A.K.); sufang.liu@tamu.edu (S.L.)

**Keywords:** overlapping orofacial pain, temporomandibular disorders, sex differences, migraine, comorbidity

## Abstract

Sexual dimorphism in temporomandibular disorders (TMDs) and their comorbidity with migraine are important phenomena observed in clinics. TMDs are the most prevalent orofacial pain conditions with jaw joint and masseter muscle dysfunction. Migraine is the predominant headache commonly associated with TMDs. Women much more often suffer from this orofacial pain than men. However, currently, there is no gender-specific therapy for such pain conditions. Understanding the pathophysiological mechanisms behind sex differences in TMDs as well as their comorbidity with migraines is essential for developing novel approaches for gender-specific treatment of TMDs and related orofacial pain comorbidity. In this review, we summarize recent research progress regarding sex differences in TMDs, focusing on the underlying mechanisms including craniofacial anatomy, hormonal regulation, and roles of opioids, transient receptor potential channels, and endocannabinoid systems. We also discuss the mechanisms of comorbid TMDs and migraine. The information covered in this review will provide mechanistic insights into sex differences in TMDs and their comorbidity with migraine, which could aid in developing effective treatment strategies for the overlapping orofacial pain condition.

## 1. Introduction

The term “orofacial pain” refers to a group of pain conditions affecting the mouth, head, neck, and face [1]. According to its origin, orofacial pain can be classified as temporomandibular disorders (TMDs), which include disorders of the temporomandibular joint (TMJ) and disorders of musculoskeletal structures (e.g., masticatory muscles), neuropathic orofacial pain (e.g., trigeminal neuralgia), and neurovascular orofacial pain (e.g., migraines) [2]. Among orofacial pain conditions, TMDs are the most prevalent and widely recognized [3], which are caused by factors that prevent the jaw muscles, bones, and TMJ from working together in harmony [2,4]. Typical signs and symptoms of TMDs include jaw discomfort, facial pain, headaches, clicking or popping of the jaw, locking of the jaw, teeth grinding, tooth sensitivity, and changes in dental occlusion [5].

TMDs originate from structures controlled by the trigeminal nerve system, including the face, head, jaw muscles, TMJ, and related tissues [2]. The trigeminal nerve plays a vital role in orofacial pain as it provides sensory innervation to the tissues in these areas associated with such pain [6]. The sensory endings of trigeminal nerve fibers (myelinated Aδ-fibers and non-myelinated C-fibers) are called nociceptors, and noxious stimuli activate them. These fibers have cell bodies in the trigeminal ganglion [7]. The nociceptive signal within the trigeminal ganglion is transmitted to the higher brain regions through the trigeminal brainstem nuclei, thereby producing pain in the orofacial regions [8]. Treating orofacial pain presents a challenge to clinicians due to its complexity and multiple contributing factors. A multidisciplinary approach is beneficial for clinicians to treat different orofacial pain conditions effectively [2]. The prevalence, intensity, duration, and frequency of orofacial pain are generally higher in women than those in men [3,9]. Most currently available pain therapies rely on drugs originally developed for other conditions, which are often ineffective for pain treatment and have poor tolerance [10]. A well-documented link exists between TMDs and primary headaches, especially migraines. Researchers have found that patients with TMDs are 2.76-times more likely to develop migraines than people without TMDs [11]. In the Orofacial Pain Prospective Evaluation and Risk Assessment (OPPERA) study, patients with TMDs have a ten-fold increase in definite migraine episodes, and, on the other hand, migraine and frequent headaches are considered significant risk factors for TMDs [12].

### 1.1. Diagnostic Criteria for TMDs

The diagnostic criteria for temporomandibular disorders (DC/TMDs) have been the international standard for TMD assessment since 2014. The DC/TMDs was developed by the Research DC (RDC)/TMD Consortium of the International Association for Dental Research and the Orofacial and Head Pain Special Interest Groups of the International Association for the Study of Pain (IASP) for both research and clinical use [13]. The DC/TMDs consists of two axes. Axis I covers physical diagnoses and is divided into pain-related TMDs and intra-articular TMDs, including 12 diagnoses [13,14]. Myofascial pain and local myalgia are excluded from the specificity and sensitivity data. The DC/TMDs offers high diagnostic accuracy for various TMD subgroups using a standardized protocol that includes history and clinical examination [13,14,15]. The RDC/TMD Axis I classifies clinical TMDs into muscle diagnoses, disk displacements, and joint disorders. The most common pain-related TMDs are myalgia, arthralgia, and headache attributed to TMDs. Intra-articular TMDs commonly include various types of disk displacement, degenerative joint disease, and subluxation [16]. Axis II includes tools for assessing psychosocial status and pain-related disability, ranging from basic screening to detailed expert evaluation [13]. The TMD pain screener is used to identify pain-related issues in any clinical setting, and further assessment is recommended for patients who were screened positive. The criteria allow for a standardized approach to TMD diagnosis and treatment, which is advantageous to both clinical practice and research [13,14].

### 1.2. The Genetic Basis of Chronic TMDs

The etiology of TMDs is multifaceted and involves a variety of risk factors that contribute to the onset and progression of the disease, which include genetic predisposition, anatomical factors, such as joint morphology and muscle attachments, and psychosocial factors, such as anxiety and stress [17,18]. Several genetic factors have been associated with TMD development and progression, including genetic variations. The onset and development of TMDs are influenced by multiple genes involved in various biological processes, including genes encoding catechol-o-methyltransferase, transient receptor potential vanilloid 1 (TRPV1), and mu-opioid receptors [19,20,21]. Additionally, other genes linked to TMD development are associated with inflammation and immune responses, including genes encoding tumor necrosis factor-alpha, interleukin-1β, Interleukin-6, and Interleukin-17A [21,22,23,24,25]. Moreover, essential biological processes, including cartilage production and bone metabolism, may also have an impact on the development of TMDs. In this context, the following genes are implicated: genes encoding Asporin linked to lumbar-disc degeneration, frizzled-related protein, and growth differentiation factor 5 [21].

Genes involved in structural functions, such as genes encoding glutathione S-transferase mu 1, C-1-tetrahydrofolate synthase, cytoplasmic, alpha-actinin-3, and methionine synthase reductase, may influence the risk of temporomandibular dysfunction [26,27,28]. Systemic diseases affecting collagen synthesis are linked to TMD development [29]. There is an association between collagen degradation failure, chronic disc inflammation, and articular surface degeneration in TMJ that causes internal derangements [30]. A disintegrin and metalloproteinase with thrombospondin motifs family is involved in disc destruction under degenerative conditions [29,31]. Matrix metalloproteinases also contribute to cartilage degradation in TMJ [28,32]. Women are more commonly affected by cartilaginous degeneration linked to the estrogen receptor 1 gene and experience chronic arthralgia twice as often, associated with osteoprotegerin and the receptor activator of nuclear factor kappa-Β ligand [33]. The estrogen alpha receptor, found in intra-articular cartilage and osteocytes, acts as an intracellular regulator and is abundant in women with TMDs. Estrogen-related receptor B encodes a protein similar to the estrogen receptor, linked to muscle and joint dysfunction and tendon degeneration [26,29]. In summary, genetic factors significantly impact the signs, symptoms, onset, and progression of TMDs, emphasizing their pivotal role in defining the course of the disorders. These insights into genetic predispositions offer valuable avenues for developing targeted treatments for TMDs.

### 1.3. Childhood Trauma’s Role in the Development of Chronic TMDs

Childhood maltreatment is widespread, often unrecognized, and underreported [34]. Childhood maltreatment is classified into four main categories by the World Health Organization (WHO): physical abuse, emotional abuse, sexual abuse, and neglect [35]. Physical abuse occurs when a caregiver intentionally harms a child, while sexual abuse involves the perpetrator engaging the child in sexual activities for his or her gratification. Child neglect includes the failure to meet a child’s basic needs, including nutrition, health, safety, education, and emotional support [34]. Childhood maltreatment is prevalent and can cause long-term physical and mental health issues, contributing to the development and progression of diseases such as TMDs and increasing the risk of developmental abnormalities [36]. A recent study reported that childhood maltreatment correlates with increased TMD severity, highlighting the need for the collaboration between dentists and mental health professionals in treating TMD patients [36]. Previous studies have suggested that individuals with TMDs are more likely to have experienced traumatic life events compared to those without TMDs. Approximately half (49.7%) of chronic TMD patients reported having experienced at least one traumatic life event [37]. The effects of psychological factors on TMDs are significant, with up to 75% of TMD patients experiencing psychological abnormalities [38]. TMD patients are more likely to experience anxiety, somatization, and depression, and psychological factors like emotional stress have greater impacts on TMDs [36,39,40]. There is a strong association between stress and increased incidence of chronic TMDs [40]. Child abuse increases the risk of chronic pain. A recent study indicates that individuals who have experienced interpersonal violence often personify chronic pain in ways reflecting past abusive experiences [41]. Given this information, it is reasonable to postulate that childhood maltreatment may contribute to emotional and psychological issues that could influence the development of TMDs. Screening for childhood trauma in TMD patients is crucial. Patients should be thoroughly evaluated and asked about any history of childhood maltreatment, with referral for psychiatric assessment as needed [41].

### 1.4. Aim of the Review

This review aims to summarize and discuss the recent research on the investigation of mechanisms that underlie sex differences in TMDs and their comorbidity with migraine. We focused on the roles of craniofacial anatomy, hormonal influences, opioid and endocannabinoid systems, and transient receptor potential (TRP) ion channels. We also explore the intriguing relationship between TMDs and migraine, discussing mechanisms for this comorbidity. Through this comprehensive review, we aim to provide mechanistic insights that will aid the future development of effective sex-specific treatments for TMDs and relevant orofacial overlapping pain conditions.

## 2. Materials and Methods

This study was a critical narrative review conducted through a comprehensive analysis of the literature. We focused on the mechanisms of sexual dimorphism in TMDs and their comorbidity with migraine, emphasizing the importance of understanding these conditions for the development of sex-specific therapies for orofacial overlapping pain.

### 2.1. Search Strategy

Relevant studies were identified through systematic searches of electronic databases, including PubMed, Scopus, Web of Science, and Google Scholar. The search covered articles published in English from January 2003 to May 2024. We also manually scanned the reference lists of relevant articles to identify additional studies.

### 2.2. Inclusion and Exclusion Criteria

We prioritized peer-reviewed research articles, including both preclinical and clinical studies, as well as review articles and systematic reviews published within the last 10 years. Studies were selected based on their relevance to TMDs and migraine comorbidity, with a particular focus on chronic TMD pain and its sex differences. Keywords used in our search strategy included temporomandibular disorder, migraine, sex differences, chronic pain, comorbidity, childhood trauma, genetic basis, ICD-11 classification, and diagnostic criteria. Articles not available in English or those lacking primary data or detailed reviews were excluded from our analysis.

### 2.3. Data Extraction

Data extraction involved summarizing the key findings, methodologies, and conclusions from each selected study. We focused on extracting information on the pathophysiological mechanisms behind sex differences in TMDs and their comorbidity with migraine.

### 2.4. Study Selection Process

A total of 126 articles were included in this review. The selection process began with a screening of titles and abstracts for relevance, followed by a full-text review of potentially eligible studies. Studies were included if they provided significant insights into the mechanisms underlying sex differences in TMDs and their comorbidity with migraine. Any discrepancies in study selection were resolved through discussion among the authors until a consensus was reached.

## 3. Sex Differences in TMDs

TMDs are the most prevalent non-dental orofacial pain condition. The epidemiological evidence consistently shows that women experience higher incidents of TMDs than men [3]. A retrospective study demonstrates that women are three times more likely to suffer from TMDs than men [42]. In addition, women are more likely to be diagnosed with significant limits in their ability to move their jaws accompanied by persistent excruciating pain and exhibit more clicking sounds at the TMJ than men [43]. In the past, it was believed that TMDs primarily affected women of a childbearing age [44]. However, recent large-scale studies in Europe and the United States show that TMD prevalence peaks between the ages of 45–64 years and gradually decreases thereafter [44]. Recently, it has been revealed that TMD patients experience lower life satisfaction and poor sleep quality, with women reporting worse sleep quality than men. These findings highlight the importance of addressing both sleep quality and life satisfaction in managing TMDs and suggest a link to sexual dimorphism in this condition [45].

In this review, we discussed sex differences in chronic pain experiences, particularly in the context of TMDs. Chronic pain, unlike acute pain that resolves with healing, persists and involves complex biological, psychological, and social factors [40]. Several organizations have developed classification systems for TMDs, for instance, TMDs are defined by the American Association for Orofacial Pain as “a group of musculoskeletal and neuromuscular conditions involving the TMJ, masticatory muscles, and associated tissues” [46]. Unfortunately, all classification systems for TMDs, whether from the past or present, share the common issue of having inherent shortcomings in fulfilling the criteria previously mentioned [14].

The 11th version of the International Classification of Diseases (ICD-11) aims to enhance the classification of chronic pain [47]. The IASP established a task force to create a consistent classification of chronic pain. This task force worked closely with the WHO, the International Headache Society, the IASP Special Interest Group on Orofacial and Head Pain, the American Academy of Orofacial Pain, and the International Network for Orofacial Pain. The classification focuses exclusively on chronic pain syndromes, defined as persistent or recurrent pain lasting longer than 3 months, and excludes acute pain [48,49]. The ICD-11, endorsed by the WHO in 2019, categorizes TMDs under different sections: “temporomandibular joint disorders” in Chapter 15 (musculoskeletal diseases) and “headache or orofacial pain associated with chronic secondary TMD” in Chapter 21 (symptoms/clinical findings). Chapter 21 also systematically classifies chronic pain into primary and secondary pain disorders [48]. The IASP’s chronic pain definition aligns with widely used criteria and covers the most relevant conditions. For headache and orofacial pain, chronicity includes attack frequency due to its episodic nature. Chronic headache is defined as lasting at least 2 h per day on more than 50% of days for over 3 months [48].

**Figure 1:**
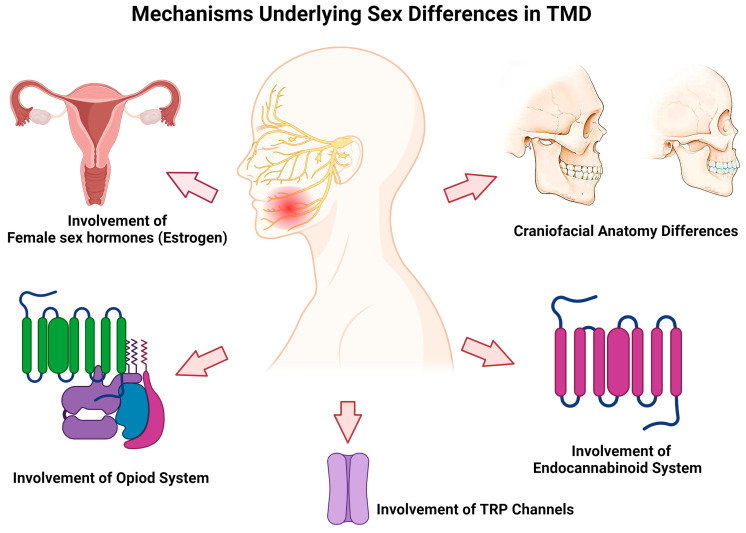
A schematic diagram showing that multidimensional factors contribute to sex difference in TMDs, including craniofacial anatomy, sex hormones, opioid and endocannabinoid systems, and TRP channels.

Here, we summarize recent studies on the underlying mechanisms for sex differences in TMDs, specifically focusing on the contribution of craniofacial anatomy, hormonal regulation, the opioid system, TRP ion channels, and endocannabinoid system (Figure 1).

### 3.1. Sexual Dimorphism of Craniofacial Anatomy and TMD Susceptibility

There are sexual dimorphisms in craniofacial sizes [50]. The craniofacial sizes of men are substantially larger than women’s [51]. Male and female mandibles differ primarily in their length, breadth, and height based on the morphological characteristics [52]. In males, the maxillary or upper jaw bones exhibit a greater length, width, and thickness compared to females. Additionally, the angle formed by the top and bottom of the mandible, or lower jaw, is less obtuse in males. This anatomical distinction is accompanied by a larger mandibular condyle or head and a deeper temporal fossa or socket [53]. These significant sex-specific differences in human mandibular sizes indicate differences in moment arm lengths of bite forces as well as potential dissimilarities in masticatory biomechanics between males and females, as the bite force magnitude is correlated with craniofacial morphology [52]. The masticatory muscles drive mandibular motion and facilitate mechanical contact between the TMJ condyle and articular disc, which influences internal joint mechanics and potentially contributes to TMDs through collagen fiber fatigue in the disc [54,55]. The mechanical functions of male and female masticatory systems are significantly different due to sex-specific differences in craniofacial morphology and muscle attachment morphometry for masseter, temporalis, lateral, and medial pterygoid muscles, with significant correlations between TMJ reaction forces and mandibular size, which may predispose females to developing TMDs [52]. Collectively, these structural variations in males and females could contribute to sex differences in TMD susceptibility [53].

### 3.2. Hormonal Impact on Sex-Specific TMD Pain

Given that 80% of patients with TMDs are women, particularly those women aged 20–40 years, questions arise regarding the underlying pathogenesis and a potential connection to the female hormonal axis [56,57]. Endogenous reproductive hormones (especially estrogens) may play a pathophysiological role in TMDs as they do in many other pain conditions (such as migraines) that affect mostly women [57,58,59]. Estrogens refer to a group of female hormones, including estrone, estradiol, and estriol, and chemically belong to the steroid family [60]. Previous studies have shown that TMD pain starts post-puberty, with a higher prevalence in women than men, decreasing post-menopause [57], which supports the potential role of estrogens in TMD pain. Estrogens initiate their biological activities by binding to estrogen receptors (ERα and ERβ) [61]. Estrogens affect inflammation and pain in both peripheral and central nervous systems. It is well-established that estrogens can directly regulate the production of inflammatory cytokines in monocytes and macrophages, which may promote inflammation and cartilage reabsorption when present in the synovium surrounding the TMJ during inflammation. Estrogens may influence TMD pain severity and predisposition, with genetic variations at ERα potentially modifying the effects [62]. Estradiol secretion is enhanced by the acute stimulation of TMJ nociceptors in a sex-dependent manner. In addition, progesterone or allopregnanolone supplements may be useful for treating estrogen-induced TMJ inflammation [63,64].

The use of estrogens significantly affects TMDs, underscoring the role of female reproductive hormones, particularly estrogens, in TMD pain [57,65]. A previous study observed that either estrogen or TMJ inflammation enhances the excitability of TMJ-innervating neurons and increases their spontaneous activity [66], and such effects of estrogen and TMJ inflammation are additive, suggesting the role of estrogen in sex-specific TMD pain [66]. Pain intensity in women with TMDs varies with the menstrual cycle, increasing before menstruation and during ovulation due to low or fluctuating estrogen levels [67]. A high estrogen level during pregnancy raises pain thresholds and improves pain inhibition [68]. Pain inhibition is more effective when estrogen levels are high in the preovulatory phase [69]. As estrogen levels fluctuate throughout a woman’s lifespan, facial pain can be induced during childbearing ages and perimenopause, while in post-menopause women, lower estrogen levels may exacerbate the degeneration of the TMJ and cause the loss of alveolar bone [58]. There is a significant contribution of estrogen signaling to the sexual dimorphism of TMDs [70]. Estrogen influences TMD pain through various mechanisms, depending on pain type, duration, and fluctuating hormone levels [71]. The estrogen receptors (ERα and ERβ) are expressed in the rat joint [72]. Genetic studies suggest that ERα polymorphisms contribute to increased TMD risk and severity, which could cause severe TMJ pain and altered mandibular dimensions in females [73]. It is well recognized that estrogen influences nociceptive processing in the trigeminal subnucleus caudalis (Vc) and the upper cervical spinal cord (C1–2 region), affecting pain and autonomic pathways, which may contribute to chronic TMJD pain, particularly in females [74]. In rats, females have a higher density of ERα-positive neurons in the superficial laminae of the spinal trigeminal nucleus caudalis and the upper cervical dorsal horn compared with males, especially during proestrus [75]. Estrogen receptors can regulate orofacial pain by modulating neuropeptide signaling in female trigeminal neurons, with a strong correlation to the oestrous cycle [76]. Specifically, estrogen receptors stimulate nociceptive responses in trigeminal neurons through galanin and neuropeptide Y [76]. Changes in neuropeptide levels within trigeminal neurons throughout the menstrual cycle could lead to more painful episodes during specific phases [76]. In a nutshell, hormones, especially estrogens, have a significant impact on pain perception, inflammation, immune system modulation, neuropeptide expression, and tissue remodeling in the TMJ, which contributes to the high incidence of TMDs in women.

### 3.3. The Opioid System and Sex Differences in TMDs

Opioid receptors in both neurons and non-neuronal cells in peripheral and central nervous systems play a vital role in analgesia [77]. Mounting evidence indicates that males and females respond differently to opioid receptor-mediated effects due to sex differences in endogenous opioid peptides and opioid receptors [78]. It is well recognized that the presence of peripheral opioid receptors in the trigeminal nerve system is potentially involved in orofacial pain modulation [77]. Opioid receptors belong to G protein-coupled receptors (GPCRs), which consist of four members: the classical μ opioid receptor (MOR), δ opioid receptor (DOR), and κ opioid receptor (KOR), along with the non-classical nociceptin/orphanin’ FQ receptor (NOR) [79]. Classical opioid receptors, sensitive to naloxone, are activated by endogenous peptides like β-endorphin, enkephalins, and dynorphins. NOR, insensitive to naloxone, is activated by nociceptin/orphanin’ FQ (N/OFQ) [80].

Bai et al. reported that sex differences in the peripheral MOR-mediated analgesia exist in an experimental model of orofacial pain, and these differences are partly mediated by changes in MOR expression, with testosterone playing a key role [81]. The findings provide insights into sex-specific MOR function and potential pharmacological pain therapies [81]. In a recent study, Fiatcoski et al. investigated the sex differences in descending control of nociception (DCN) responses in rats with chronic orofacial pain, where female rats showed a reduction in DCN compared to male rats, and chronic orofacial pain induction caused a significant loss of DCN responses in female rats [82]. More importantly, DCN loss in female rats can be prevented by pretreatment with nor-BNI, a specific KOR antagonist. These results indicate sex differences in DCN responses and a female-specific impairment following chronic orofacial pain. The inhibition of KOR may be a promising approach to treating chronic orofacial pain, especially in females [82]. Similarly, Saloman et al. investigated the responses of male and female rats to a DOR agonist in an acute orofacial muscle pain condition. Both sexes showed reduced hypersensitivity to the treatment with the DOR agonist, but females required a higher dose of the DOR agonist [83]. In males, blocking ATP-sensitive K^+^ channels, a downstream target of opioid receptor signaling, abolished DOR-mediated effects [83]. The expression of the ATP-sensitive K^+^ channel is higher in males than in females [84]. These studies indicate that there are sex differences in pain relief mediated by DOR and sex-specific variations in ATP-sensitive K^+^ channels in orofacial muscle pain [83,84]. Taken together, these studies underscore the vital role of different opioid receptors in orofacial pain, with potential sex differences in the underlying signaling.

### 3.4. The Endocannabinoid System and Sex Differences in TMDs

The endocannabinoid system is a complex biological network of cannabinoid receptors, endogenous ligands, and enzymes. It regulates pain, immunomodulation, inflammation, and other physiological functions. CB1 and CB2, the two most studied cannabinoid receptors, belong to the GPCR superfamily and have seven transmembrane-spanning domains [85]. The CB1 receptor modulates trigeminal neurons and is expressed in brain regions related to orofacial pain, such as the trigeminal nucleus caudalis and spinal trigeminal tract. In the peripheral nervous system, the CB1 receptor triggers antinociceptive effects by regulating nociception primarily in sympathetic nerve terminals, trigeminal ganglions, and sensory nerve endings [86]. The CB2 receptor, primarily in the immune system and microglia, can regulate immune cell proliferation and chemotaxis. According to recent studies, the CB2 receptor may be involved in neurological processes like neuroinflammation, drug addiction, and nociception [85,87].

It is vital to understand how the endocannabinoid system functions differently in each sex and whether there is sex-dependent cannabis analgesia [88]. There are sex differences in the cannabis response, with females being more susceptible to Delta-9-tetrahydrocannabinol [89]. Sex differences in cannabis reactions may be due to direct interactions between the endocannabinoid system and sex hormones [89]. Niu et al. reported the effects of gonadal hormones on the peripheral CB1R system in a rat orofacial pain model [90], where complete Freund’s adjuvant was injected into the masseter muscle, the sex difference in cannabinoid-produced peripheral antinociceptive effects was observed, and more importantly, a CB1-selective agonist showed more potent efficacy in reducing allodynia in male rats compared to female rats [90]. In trigeminal ganglia under inflammatory conditions, testosterone, not estradiol, was required for CB1 receptor modulation, which indicates the potential sex differences in cannabinoid effects [90]. Similarly, Lee et al. reported that inflammation upregulated CB1 receptor expression in male, but not female, trigeminal ganglia via testosterone-dependent mechanisms [91]. Testosterone activates *Cnr1* gene transcription via the androgen receptor in response to cytokine stimulation. These studies suggest sex differences in the peripheral cannabinoid system [91].

### 3.5. TRP Ion Channels and Sex Differences in TMDs

TRP ion channels, particularly the six subfamilies including vanilloid (TRPV), ankyrin (TRPA), melastatin (TRPM), canonical (TRPC), polycystin (TRPP), and mucolipin (TRPML), are key contributors to orofacial pain. These non-selective cation channels conduct Ca^2+^ and act as molecular sensors for various stimuli, converting them into electrical signals that are sent to the central nervous system [92,93]. Recently, a study reported that TRPA1 and TRPV1 ion channels play key roles in TMD pain [94]. In mouse models of TMD pain induced by TMJ inflammation and masseter muscle injury, knockout or inhibitions of TRPA1 and TRPV1 reduced pain [94]. The upregulation of TMEM100, TRPA1, and TRPV1 in trigeminal ganglion neurons after TMD pain suggests TMEM100’s role in regulating TRPA1 within the TRPA1-TRPV1 complex, making it a potential target for TMD pain treatment [94]. TRPV1 contributes to sexual dimorphism in TMD pain, with estradiol potentially upregulating its expression in TMJ synovium and central neural structures like the hippocampus [95]. In a rat model of TMJ arthritis, complete Freund’s adjuvant (CFA) was injected into the TMJ to induce inflammation [96]. Female rats underwent bilateral ovariectomies or sham procedures and received daily subcutaneous injections of 17-β-estradiol for 12 days. This treatment led to a dose-dependent upregulation of TRPV1 and nerve growth factor in TMJ synovium [96]. Capsazepine, a TRPV1 antagonist, significantly mitigated TMJ allodynia induced by CFA injection in estradiol-treated rats, underscoring TRPV1’s pivotal role in the sexual dimorphism of TMD pain [96].

Table 1 illustrates the underlying mechanisms for sex differences in TMDs.

## 4. TMDs and Comorbid Pain Conditions

The term “comorbidity” refers to an additional ailment that coexists with temporal relation to the ailment undergoing examination [97]. The 2011 Institute of Medicine report titled “Relieving Pain in America” highlights chronic pain’s significance and magnitude to the American public. There has been increasing recognition that certain chronic pain conditions tend to coexist, with women experiencing them more frequently than men [98]. According to the National Institute of Health and the US Congress, coexisting pain conditions encompass a wide range of disorders co-aggregating together, including TMDs, migraines, chronic tension-type headaches, fibromyalgia, irritable bowel syndrome, vulvodynia, chronic fatigue syndrome, interstitial cystitis, endometriosis, and chronic lower back pain. Chronic overlapping pain conditions refer to these conditions collectively [99]. It is well-established that TMDs have been linked to a multitude of pain conditions, including headaches, fibromyalgia, chronic back pain, irritable bowel syndrome, and chronic fatigue syndrome [100]. In theoretical terms, there are several reasons why disease symptoms may coexist, such as shared etiologic pathways [101]. The predominant pathway is central sensitization, which is a result of increased synaptic effectiveness amplifying nociceptive and sensory stimuli [102]. Based on the high degree of comorbidity with other disorders, the most plausible pathologic explanation for centralized pain in TMDs is because of imbalances in neurotransmitters, which, contribute to comorbid pain conditions and could disrupt sleep, memory, and other functions. A persistent pain response could result from either an increase in synaptic transmission in the pronociceptive pathways, a decrease in synaptic transmission in the antinociceptive pathways, or a combination of these pathways [103]. Here, we focus specifically on the comorbidity of TMDs with migraine and the underlying mechanisms.

### 4.1. The Link between TMDs and Migraines

Migraine is the primary headache that has the highest prevalence among patients with TMDs [104]. A migraine is characterized by recurrent attacks of throbbing pain on one side of the head, nausea, vomiting, photophobia, and phonophobia [105]. A recent study indicates that cranial autonomic symptoms are associated with frequent episodic tension-type headaches as well as migraines and trigeminal autonomic cephalalgias, and this finding underscores the need for careful diagnosis, given the potential overlap of symptoms in various headache disorders [106]. Statistically, most headache disorders are secondary, meaning that they are comorbid symptoms of another condition [107]. The co-occurrence of migraine and TMDs suggests a potential correlation between their underlying causes [104]. A recent investigation reported that the TMD group experienced a much higher prevalence of headaches (72%) compared to the control group (31%), and migraine notably accounts for 55% of headaches in patients with TMDs [11]. A previous study that investigated the relationship between TMDs and migraine development observed a substantial correlation (odd ratio = 4.1) between the frequency of TMD symptoms and headache frequency, indicating that TMDs may be a risk factor for chronic migraine [108]. The prevalence of migraine among TMD patients is up to 58%, especially in women with a myogenic TMD [109]. A variety of multidimensional factors are linked to an increased risk of comorbid TMDs and migraines. In the following sections, we discuss various components contributing to increased risk factors for the comorbidity of TMDs with migraines.

### 4.2. Craniofacial Neuroanatomy and Clinical Connections in Comorbid Migraines and TMDs

The trigeminal nerve plays a crucial role in both migraine and TMDs. The ophthalmic branch (V1) of the trigeminal nerve often mediates migraines, which can spread to the facial areas innervated by the maxillary (V2) and mandibular (V3) branches of the trigeminal nerve. TMD symptoms are linked to the V3 branch of the trigeminal nerve. [110,111]. It is unusual to experience isolated facial pain limited to the V2- or V3-innervated face, a condition recently recognized by the International Classification of Orofacial Pain [112]. Both migraine and TMD pain conditions involve nociceptive signals that converge at the caudal nucleus of the spinal trigeminal nuclei. They share common pain processing pathways, including the limbic system, brainstem nuclei, sensory cortex, and thalamus [113]. Neurons in the trigeminal nucleus caudalis receive inputs from various sources and integrate them, sending the combined signals to the thalamus and onward to the somatosensory cortex as well as limibic cortices (anterior cingulate cortex and insular cortex). This convergence point suggests that migraine and TMDs may influence each other [111]. TMD pain can spread throughout the orofacial and cranial regions. Because the muscles of mastication, the TMJ, and the head have a close anatomical relationship, migraine patients can experience TMD pain with the highest incidence [114]. TMDs and migraine share similar characteristics of head and/or facial pain and are more prevalent in women, particularly those of a childbearing age. The two disorders often overlap, resulting in misdiagnosis or underdiagnosis. During migraine onset, patients frequently experience spontaneous pain in the jaw muscles, teeth, cheek, and around the ears, which can be mistaken for dental, sinus, or TMD-related pain, leading to inappropriate treatment, including unnecessary tooth extractions that do not relieve the pain [11]. In a nutshell, the comorbid migraine and TMD pain condition can be partly due to craniofacial neuroanatomy, which involves brainstem alterations and trigeminal nerve sensitization.

### 4.3. Peripheral and Central Sensitizations in Comorbid Migraines and TMDs

The shared nociceptive pathways in the trigeminal nerve system may underlie the overlapping of painful TMDs and migraines via peripheral and central sensitizations [115]. The V1 (ophthalmic), V2 (maxillary), and V3 (mandibular) branches of the trigeminal nerve are principally responsible for innervating the craniofacial tissues. Specifically, the V1 branch innervates the supraorbital tissues, meninges, and cornea, the V2 branch innervates the infraorbital skin and upper lip, and the V3 branch innervates the lower jaw and lip [116]. The sensory cortices receive action potentials that are triggered by the activation of different types of afferent endings, and these action potentials provide sensory information about stimulus location, quality, intensity, and duration. More importantly, nociceptors become more excitable following inflammation or injury, resulting in continued activity and increased sensitivity, a condition known as peripheral sensitization [117]. Several chemical mediators, such as glutamate, γ-aminobutyric acid, serotonin, noradrenaline, and neuropeptides, are involved in the peripheral sensitization of nociceptive signals. Sex differences may exist in the peripheral actions of these mediators, and nociceptive afferents can undergo phenotypic adaptations or switches [116]. Persistent peripheral pain input can lead to central sensitization, where central pain pathways become more excitable, causing hyperalgesia and allodynia [118]. Both TMDs and migraine often induce myofascial pain with trigger points, which can produce migraine episodes through both central-to-peripheral and peripheral-to-central mechanisms. Moreover, they can mutually provoke each other via the cross-excitation of trigeminal nerve branches, with anatomical connections between V1, V2, and V3 [11]. In summary, peripheral and central nociceptive mechanisms, myofascial trigger points, as well as trigeminal ganglion cross-excitation are linked to comorbid migraine and TMDs. Understanding these mechanisms may aid us in developing targeted therapies for this comorbidity.

### 4.4. Role of the Calcitonin Gene-Related Peptide (CGRP) in Comorbid Migraines and TMDs

The neuropeptide CGRP is known as an integral component of migraine pathogenesis and it also plays an important role in TMDs. Its involvement in both conditions can provide valuable insights into their common mechanisms and potential therapy development [119]. It has been reported that patients with TMDs have higher levels of CGRP in synovial tissues than healthy controls, which positively correlates with the intensity of TMJ pain [120]. CGRP signaling contributes to pain mechanisms in both peripheral and central nervous systems. Trigeminal nuclei, peripheral nerve endings, trigeminal ganglion cells, and afferent projections express CGRP receptors [119]. The CGRP is released from trigeminal nerve fibers and is believed to play a vital role in neurogenic inflammation and migraine. Since CGRP receptors are widespread in the trigeminal nerve system, an increase in CGRP level under TMD pain conditions could lead to migraine development. In contrast, the elevated level of CGRP during migraine attacks may aggravate TMD symptoms [11]. The role of CGRP in the comorbid migraine and TMDs has been studied recently with a combination approach, in which the injection of complete Freund’s adjuvant into the masseter muscle can produce migraine-like neuronal response and exacerbate somatosensory-evoked cranial hypersensitivity [119]. In a nutshell, these studies indicate that targeting the CGRP pathway could be developed into an effective monotherapy for this overlapping pain condition.

### 4.5. Role of Trigeminal Dynorphin in Comorbid Migraines and TMDs

Dynorphin, an endogenous opioid neuropeptide, plays multiple roles in pain, addiction, and mood regulation. These substance-produced effects are mediated by the KOR, a GPCR [121]. The prodynorphin (*Pdyn*) gene encodes several opioid neuropeptides, including dynorphin A (dynA), dynorphin B (dynB), neoendorphins, and inactive fragments [109]. dynA is involved in chronic pain development and maintenance, whereas dynB and neoendorphin contribute to post-sensory antinociception [122,123]. Increased dynorphin expression is a potential mechanism for the transition from acute to chronic pain [124]. In addition, dynA has been reported to stimulate kinin receptors, potentially contributing to the maintenance of orofacial pain [125]. In our recent study [109], we reveal the female-specific role of trigeminal dynorphin in comorbid TMDs and migraine-like pain. We developed a novel animal model to study orofacial pain comorbidity by combining masseter muscle tendon ligation with the systemic injection of nitroglycerin [126]. Interestingly, we found that pre-existing myogenic TMDs induced by masseter muscle tendon ligation enabled a subthreshold dose of nitroglycerin to produce migraine-like pain in mice [126]. By RNA sequencing followed by real-time quantitative polymerase chain reaction confirmation, we identified *Pdyn* expression is significantly increased in the trigeminal nucleus caudalis of female, but not male, mice [109]. Moreover, we further found that the chemogenetic inhibition of *Pdyn*-expressing neurons or microinjection of antidynorphin antiserum in the trigeminal nucleus caudalis diminishes the overlapping migraine and myogenic TMD pain in female mice but not in male mice [109]. Therefore, targeting trigeminal dynorphin may offer therapeutic potential for treating comorbid migraines and TMDs [109].

Table 2 illustrates the mechanisms underlying comorbidity between TMDs and migraine.

## 5. Conclusions and Future Directions

In this review, we primarily focused on the mechanisms underlying sex differences in TMDs and their comorbidity with migraine. TMDs refer to chronic orofacial pain conditions that result in jaw joint and muscle dysfunction, leading to symptoms like muscle pain, joint pain, clicking, and reduced jaw mobility. Patients with TMDs exhibit sex differences, with a high prevalence in women. The factors contributing to sex differences in TMDs include craniofacial anatomy, hormonal regulation, and opioid and endocannabinoid systems. Identifying these factors will aid the future development of sex-targeted interventions and improve the clinical management of such pain. Additionally, this review sheds light on the intertwined relationship between TMDs and migraine, with peripheral and central nociceptive mechanisms, myofascial trigger points, trigeminal ganglion cross-excitation, CGRP, and trigeminal dynorphin working together to play crucial roles in this comorbidity. Therefore, these shared pathophysiological mechanisms can be targeted to develop novel therapies for comorbid migraine and TMD pain conditions. In the future, longitudinal studies should focus on how sexual dimorphism affects the onset, progression, and outcome of TMDs. These studies can provide insights into sex-specific risk factors and treatment responses in TMDs. Understanding molecular, genetic, and neurophysiological mechanisms driving sex differences in TMDs requires mechanistic investigations. A multifaceted approach is also needed to understand the mechanisms underlying the comorbidity between TMDs and migraines. For the comorbid pain condition, practitioners should use a nuanced diagnosis and treatment strategy that considers overlapping symptoms and sex-specific variations in pain perception and treatment responses. By tailoring treatment plans and emphasizing patient education on the sex-specific aspects of TMDs and migraines, therapeutic outcomes and patient satisfaction can be enhanced.

## 6. Limitations

Our review has several limitations. Firstly, excluding non-English studies may introduce publication bias. Our findings may also be limited in generalizability due to the variability in study designs and methodologies among the included articles. While we reviewed a wide range of literature on the sexual dimorphism of TMDs, the depth of analysis varied between studies. It would be helpful if future reviews can use stricter inclusion criteria and meta-analytic approaches to quantitatively synthesize the findings. Furthermore, the use of preclinical models that may not fully replicate human conditions may limit the applicability of the results. This limitation will be addressed by the application of rigorous quality assessment tools.

## Figures and Tables

**Table 1:** Mechanisms underlying sex differences in TMDs.

Mechanisms	Findings	Sex Differences	Clinical Implications	References
Craniofacial Anatomy	Men have larger craniofacial sizes and mandibles compared to women.	Male mandibles are longer, broader, and taller; female mandibles have more obtuse angles.	These differences affect bite force and masticatory biomechanics, possibly predisposing females to TMDs.	[50,51,52,53,54,55]
Hormonal Impact	Estrogens play a significant role in TMDs, influencing pain and inflammation in peripheral and central nervous systems.	Higher prevalence of TMDs in women, especially during reproductive years; pain intensity varies with the menstrual cycle.	Estrogen-based therapies may be effective; hormonal regulation could reduce TMD pain.	[56,57,58,59,60,61,62,63,64,65,66,67,68,69,70,71,72,73,74,75,76]
Opioid System	Opioid receptors mediate analgesia, with sex-specific differences in endogenous opioid peptides and receptor responses.	Females may have reduced descending control of nociception and require higher doses of certain opioid agonists.	Sex-specific opioid receptor targeting and KOR inhibition may improve pain management in females.	[77,78,79,80,81,82,83,84]
Endocannabinoid System	CB1 and CB2 receptors modulate pain and inflammation, with sex-specific responses to cannabinoids.	Females are more susceptible to THC effects; CB1 receptor modulation is testosterone-dependent in males.	Personalized cannabinoid-based therapies considering sex differences may enhance efficacy.	[85,86,87,88,89,90,91]
TRP Ion Channels	TRPA1 and TRPV1 are crucial for TMD pain; estradiol may upregulate TRPV1 expression in TMJ synovium.	TRPV1 upregulation in females is influenced by estradiol; TRPA1-TRPV1 complex regulation differs by sex.	Considering hormonal influences, targeting TRPV1 and TRPA1 channels may reduce TMD pain in females.	[92,93,94,95,96]

**Table 2:** Mechanisms for TMD and migraine overlapping pain.

Mechanisms	Description	References
Craniofacial Neuroanatomy	The trigeminal nerve branches (V1, V2, and V3) play a crucial role, with nociceptive signals converging at the spinal trigeminal nuclei, leading to shared pain pathways for TMDs and migraines.	[110,111,112,113,114]
Peripheral Sensitization	Increased nociceptor excitability due to inflammation or injury, involving mediators like glutamate and serotonin, contributes to TMD and migraine overlapping pain.	[115,116,117]
Central Sensitization	Persistent peripheral pain input leads to central sensitization, making central pain pathways more excitable, common in both TMDs and migraines.	[11,118]
Myofascial Trigger Points	Trigger points in masticatory muscles can provoke migraine episodes and vice versa, through central-to-peripheral and peripheral-to-central mechanisms.	[11]
Trigeminal Ganglion Cross-Excitation	Cross-excitation between trigeminal nerve branches (V1, V2, and V3) allows pain in one branch to provoke pain in another, contributing to overlapping pain in TMDs and migraines.	[11]
Role of CGRP	CGRP released from trigeminal nerve fibers plays a key role in neurogenic inflammation. Elevated CGRP levels in TMDs can lead to migraines, and vice versa.	[119,120]
Role of Trigeminal Dynorphin	Increased dynorphin expression in the trigeminal nucleus caudalis, specifically in females, is linked to TMD and migraine overlapping pain. Targeting dynorphin may offer therapeutic potential.	[109]

## Data Availability

Not applicable.
